# Anterior Dislocation of the Sternoclavicular Joint – A Case Report

**DOI:** 10.7759/cureus.20974

**Published:** 2022-01-05

**Authors:** Ashutosh Mohapatra, Priyam Choudhury

**Affiliations:** 1 Orthopaedics, Mohapatra Fracture and Accident Hospital, Mumbai, IND; 2 Diagnostic Radiology, Mayo Clinic, Jacksonville, USA

**Keywords:** anterior, closed reduction, left upper limb, sternoclavicular joint, reducible dislocation

## Abstract

Dislocation of joints in the human body is a common occurrence, with the upper limb being commonly involved in most dislocations. Despite the common occurrence of upper limb dislocations, sternoclavicular dislocation is rare, comprising just 1% of all dislocations seen. This is attributed to a combination of restricted range of movements and strong ligaments around it. A thorough search of literature revealed a handful of cases of sternoclavicular dislocation. We report a case of a 35-year-old firefighter who presented to us an hour after his injury. He was diagnosed with anterior dislocation of the left sternoclavicular joint and was successfully managed by the closed reduction maneuver. We present this case to highlight the rarity of this unusual dislocation and also shed light on its etiopathogenesis and current management trends.

## Introduction

Due to the presence of strong ligaments around it, sternoclavicular joint (SCJ) dislocation often requires a massive amount of force to cause it. SCJ dislocation is a rare injury that is classified into anterior and posterior dislocations. Anterior SCJ dislocations account for almost 3% of all shoulder girdle injuries but only 1% of all joint dislocations [1-3]. Anterior dislocation is nine times more frequent than posterior dislocation [1]. Posterior SCJ dislocation demands an accurate and timely diagnosis due to the presence of important anatomical structures like the trachea, esophagus, nerves, and great vessels. A missed diagnosis can be fatal [4]. A rapid diagnosis followed by efficient treatment is needed to avoid future complications [5]. We report the case of a 35-year-old firefighter who presented to us with anterior SCJ dislocation and was successfully treated with closed reduction. A brief literature review of SCJ dislocation is presented with regard to its mechanism of injury, presentation, diagnosis, and management.

## Case presentation

**Figure 1:**
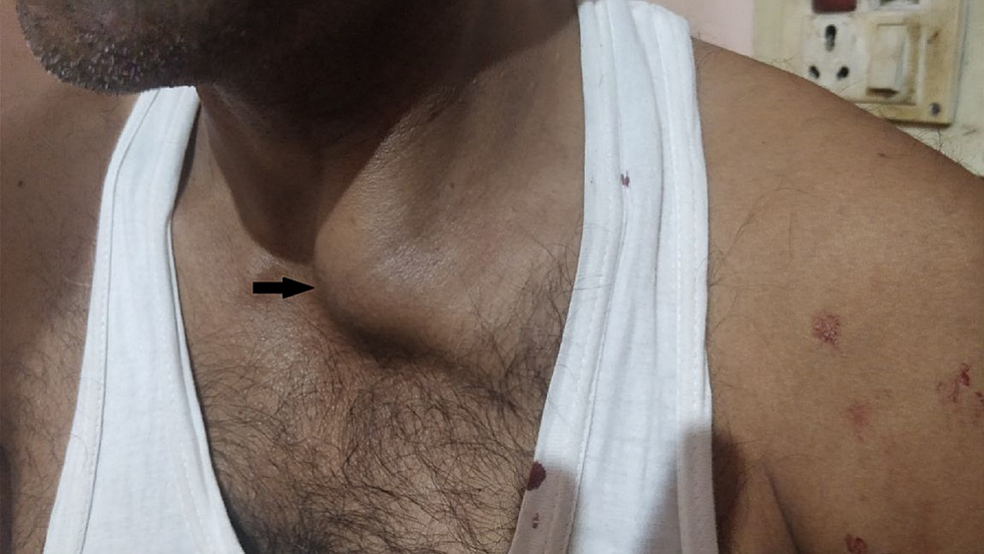
Clinical picture showing the prominent bump just lateral to the sternum (black arrow).

**Figure 2:**
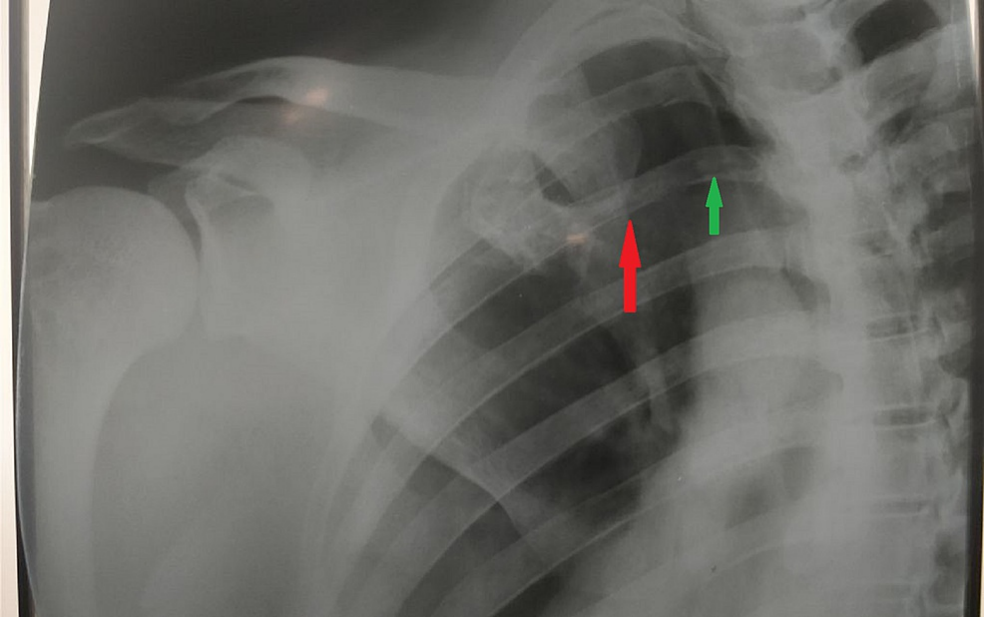
Digital radiograph showing the dislocation of the sternoclavicular joint with separation between the medial end of clavicle (red arrow) and sternum clearly visible (green arrow).

A 35-year-old man presented to our emergency department (ED) with complaints of pain and swelling in the upper portion of his left chest and left hand. A firefighter by profession, he had sustained a fall from approximately a height of 4 meters while on active duty. Almost immediately, he complained of severe pain and swelling. He was brought to the hospital an hour after his fall and was conscious and oriented with no neurovascular deficit. On examination, there was localized tenderness along with a prominent palpable bump just lateral to the sternum on the left side (Figure 1). We suspected a fracture of the sternal end of the left clavicle or an anterior dislocation of the left SCJ. He had also sustained a contused lacerated wound (CLW) of 2 mm x 2 mm on the dorsal surface of his left thumb. A series of trauma radiographs were carried out, which confirmed our suspicion of anterior dislocation of the left SCJ (Figure 2).

**Figure 3:**
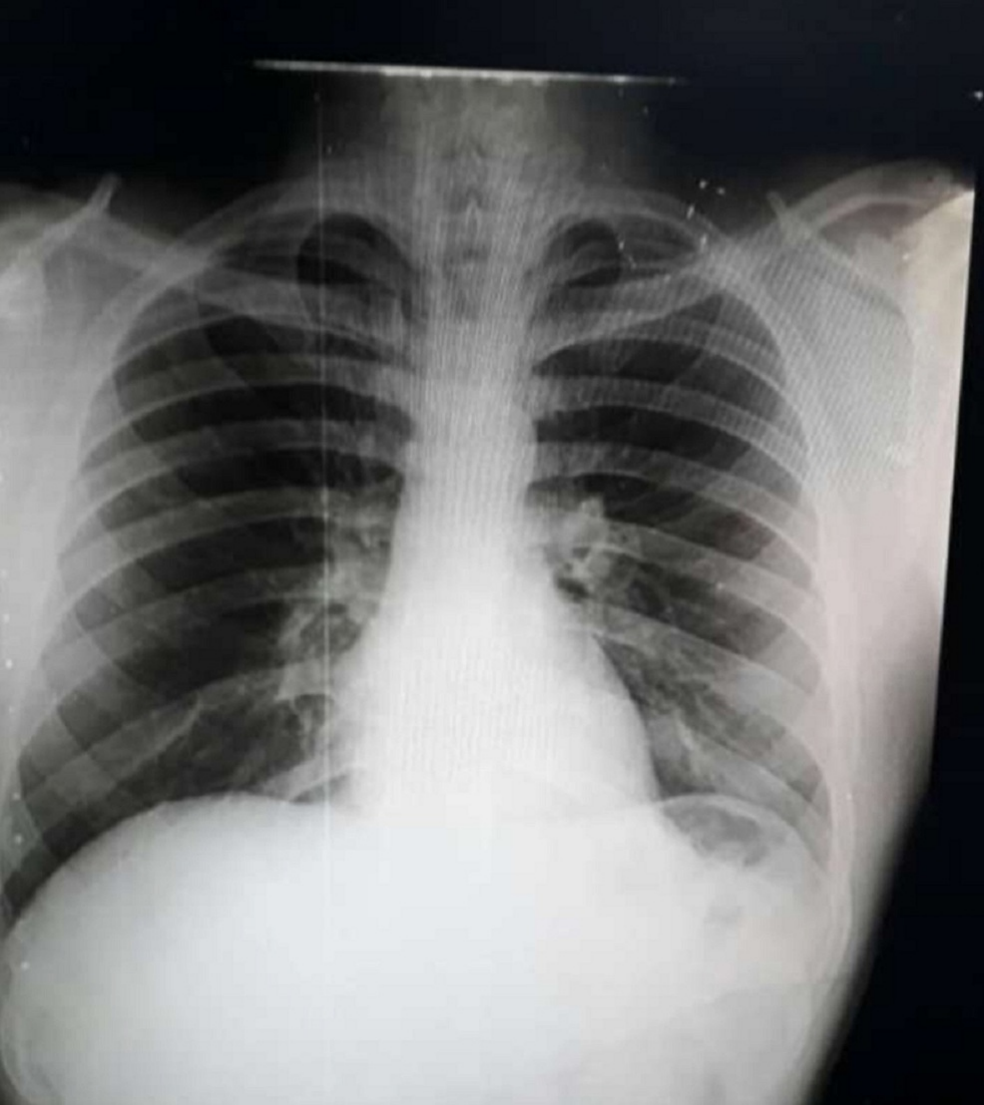
Digital radiograph of the chest showing successful maintenance of reduction of the left sternoclavicular joint.

A radiograph of the left hand revealed a displaced transverse fracture of the shaft of the first metacarpal of the left hand. There was no other osseous deformity seen. After thorough discussion with the relatives, a decision to reduce the SCJ dislocation under sedation was made. Under general anesthesia, the patient was lying supine with a bolster firmly placed between his shoulders. The upper limb was then abducted to 90 degrees with traction applied to it in neutral flexion and direct pressure applied over the sternal end of the clavicle. Successful reduction of the SCJ dislocation was confirmed clinically as well as under fluoroscopy. The first metacarpal shaft fracture was fixed with open reduction and internal fixation using a lag screw (10 mm x 2 mm). The CLW was thoroughly debrided. A polysling was applied to the left upper limb for 4-6 weeks, and the patient was called for fortnightly follow-up. At four-week follow-up, the patient was fine with the maintenance of the reduction in alignment and negligible tenderness at the injury site (Figure 3).

## Discussion

The shoulder comprises four joints: the glenohumeral joint, the scapulothoracic joint, the SCJ, and the acromioclavicular joint [6]. The SCJ has an extremely limited range of motion (ROM) of 35° forward/backward and 45° forward. Because of these minute degrees of ROM, the SCJ is a reasonably stable joint [7]. The posterior and anterior sternoclavicular ligaments, costoclavicular ligament, interclavicular ligament, and the strong joint capsule that surround the SCJ provide it with durable stability. Hence, traumatic SCJ dislocation is a very unusual injury [4]. The anterior and posterior dislocations depend upon the displacement of the sternal clavicle end over the sternum [5]. Posterior dislocation of the SCJ occurs if there is application of force directly over the anteromedial aspect of the clavicle or if the posterolateral shoulder undergoes an indirect force thereby forcing the sternal clavicle posteriorly. Meanwhile, anterior dislocation is usually caused by a lateral compressive force to the shoulder girdle which results in the posterior capsule being spared but the anterior capsule and frequently a portion of the costoclavicular ligament being ruptured [1]. The prerequisite for causing SCJ dislocation is a powerful force of vector, which can only be seen during high-energy trauma like road traffic accidents (RTA), sporting accidents, or falls from height [3]. Previous literature also mentions RTA and sporting activities like motocross racing as major causes [3-5,7-9]. Heavy activity in the form of bench press in the gym has also been reported [10]. Our patient remembers the exact incidence of events of the injury in which he jumped from a height of approximately 4 meters followed by a wall collapsing on him. This must have caused the generation of powerful force of vector required.

Patients with anterior dislocations of the SCJ clinically present with a painful lump just lateral to the sternum. Utmost precaution needs to be taken to ascertain whether this is a true SCJ dislocation or a fracture of the sternal clavicle. If the patient is under 25 then the possibility of a physeal injury should also be considered [11]. The diagnosis may be obvious from the findings of a clinical examination, which reveal local tenderness and prominence of the sternal end of the clavicle. In some patients, elevation of arm can produce a click along with a palpable subluxation. Sometimes, the physical findings are subtle, and diagnosis will depend on imaging studies. But the imaging studies highly depend on the positioning of the limb. Visualization of the SCJ on plain radiographs can be technically challenging due to the anatomical complexity of the region. The view of choice for SCJ dislocation is the ‘serendipity’ view, which shows asymmetry in the sternal ends of the clavicles [3,10]. Anterior dislocation is seen as a superiorly displaced medial end of the dislocated clavicle while posterior dislocation presents as inferiorly displaced medial end of dislocated clavicle [1]. Computed tomography (CT) scan with three-dimensional reconstruction has become the choice of imaging in doubtful SCJ dislocations [4].

There has been an ongoing debate about whether anterior dislocation warrants reduction. This is based on the theory that persistent anterior clavicular prominence causes no significant functional difficulties, and that recurrent, and even irreducible, anterior dislocations are usually tolerated without significant sequelae or complications [10]. However, closed reduction is successful in only 38% of attempts [12], and despite successful closed reduction, residual instability often remains. If closed reduction does not work, then open treatment should be considered. It has been reported that closed reduction has poor results with a high failure rate of maintaining reduction and requires a subsequent second operation [13]. However, if the closed reduction is a success, it has similar functional outcomes to the open reduction [14]. We explained to our patient and his relatives about these scenarios of management. They took a decision to proceed with the closed reduction maneuver and the dislocation was reduced as described above. 

## Conclusions

This case report describes a case of an anterior SCJ dislocation with no other osseous injury. SCJ dislocations are rare and should be investigated properly. Though the diagnosis of anterior SCJ dislocation is obvious with its clinical finding, a high degree of suspicion and astute clinical acumen are also needed since it can be missed in the setting of polytrauma. If SCJ dislocation presents within eight to 10 days of injury, it can be reduced successfully. Open reduction should be carried out for unsuccessful cases of relocation or for re-dislocation cases.

## References

[1] Morell DJ, Thyagarajan DS (2016). Sternoclavicular joint dislocation and its management: a review of the literature. World J Orthop.

[2] Renfree KJ, Wright TW (2003). Anatomy and biomechanics of the acromioclavicular and sternoclavicular joints. Clin Sports Med.

[3] Yi JW, Kim DH, Heo YM, Jun JB (2016). Bilateral sternoclavicular joint dislocation due to sternal fracture: is it a dislocation or a separation?. Arch Orthop Trauma Surg.

[4] Wang H, Wang C, Ruan J, Wu W (2019). Asymmetrical bilateral sternoclavicular joint dislocation combined with bilateral clavicular fracture: a case report. Medicine (Baltimore).

[5] Khalid N, Elbeshbeshy A, Alsaleh KA, Al-Ahaideb A (2013). Anterior sternoclavicular dislocation associated with clavicular fracture: a case report and review of the literature. Eur J Orthop Surg Traumatol.

[6] Kharat A, Maheshwari S, Choudhury P, Mohapatra A (2018). Double trouble!!! An unusual presentation of cervical cord herniation and medial end clavicle non-union in a single patient. BMJ Case Rep.

[7] Ogawa T, Masuya M, Onishi S, Iwabuchi S, Yoshii Y, Hirano A, Yamazaki M (2020). Positional anterior sternoclavicular joint dislocation in the acceleration phase of throwing: a case report. JSES Int.

[8] Terra BB, Rodrigues LM, Pádua DV, Martins MG, Teixeira JC, De Nadai A (2015). Sternoclavicular dislocation: case report and surgical technique. Rev Bras Ortop.

[9] Yadav S, Khanna V, Mukherjee S (2019). Ipsilateral sternoclavicular joint anterior dislocation with fracture of the mid shaft of the clavicle. J Clin Orthop Trauma.

[10] Hautala GS, Kamineni S (2019). Recurrent anterior sternoclavicular joint subluxation: long-term implant-related recurrence. JSES Int.

[11] Tepolt F, Carry PM, Heyn PC, Miller NH (2014). Posterior sternoclavicular joint injuries in the adolescent population: a meta-analysis. Am J Sports Med.

[12] Gerich T, Hoffmann A, Backes F, Duinslaeger AD, Seil R, Pape D (2019). Anterior buttress plate is successful for treating posterior sterno-clavicular dislocation. Knee Surg Sports Traumatol Arthrosc.

[13] Eskola A, Vainionpää S, Vastamäki M, Slätis P, Rokkanen P (1989). Operation for old sternoclavicular dislocation. Results in 12 cases. J Bone Joint Surg Br.

[14] Kirby JC, Edwards E, Kamali Moaveni A (2015). Management and functional outcomes following sternoclavicular joint dislocation. Injury.

